# Increased HIF-2α and CD105 (endoglin) expression is associated with poor differentiation in pheochromocytomas and paragangliomas

**DOI:** 10.3389/fendo.2026.1915433

**Published:** 2026-09-04

**Authors:** Tuğba Günler, İlker Çordan

**Affiliations:** 1 Department of Pathology, Hamidiye School of Medicine, University of Health Sciences, Konya City Hospital, Konya, Türkiye; 2 Department of Internal Medicine, Division of Endocrinology and Metabolism, Hamidiye School of Medicine, University of Health Sciences, Konya City Hospital, Konya, Türkiye

**Keywords:** angiogenesis, CD105, endoglin, GAPP, HIF-2α, paraganglioma, pheochromocytoma, tumor differentiation

## Abstract

**Objective:**

Reliable biomarkers reflecting tumor biology and histopathological risk status in pheochromocytomas and paragangliomas (PPGL) remain limited. In this study, the expression of biomarkers associated with hypoxia, angiogenesis, and proliferation was evaluated, and their associations with histopathological differentiation in PPGL were investigated.

**Methods:**

A total of 35 tumor foci from 31 patients with PPGL who were surgically treated were retrospectively examined. Expression levels of hypoxia-inducible factor-2 alpha (HIF-2α), vascular endothelial growth factor (VEGF), CD105 (endoglin), Ki-67, and succinate dehydrogenase subunit B (SDHB) were assessed using immunohistochemical methods. Associations between these markers and clinicopathological characteristics, Pheochromocytoma of the Adrenal Gland Scaled Score (PASS), and Grading System for Adrenal Pheochromocytoma and Paraganglioma (GAPP) scores were analyzed.

**Results:**

HIF-2α expression, CD105 microvessel density (MVD) score, and Ki-67 proliferation index were significantly higher in poorly differentiated tumors compared with moderately/well-differentiated tumors (p<0.05 for all). In contrast, VEGF expression did not differ significantly between the groups. The GAPP score showed positive correlations with HIF-2α (r=0.48; p<0.01), CD105 (r=0.54; p<0.001), and Ki-67 (r=0.77; p<0.001). In univariate analyses, bilateral disease, HIF-2α expression, CD105 MVD score, and loss of SDHB expression were associated with higher GAPP scores. However, in multivariable regression analysis, only CD105 was independently associated with the GAPP score (β=0.04; p=0.026).

**Conclusion:**

Increased expression of CD105, HIF-2α, and Ki-67 was associated with poorer histopathological differentiation in PPGL. Among the biomarkers evaluated, only CD105 showed an independent association with the GAPP score, suggesting that tumor-associated neoangiogenesis may contribute to histopathological differentiation in PPGL. These findings support further investigation of CD105 as a potential biomarker for histopathological risk assessment. However, larger studies with long-term clinical outcome data are needed to establish its clinical prognostic value.

## 1 Introduction

Pheochromocytomas and paragangliomas (PPGL) are rare neuroendocrine tumors arising from the adrenal medulla or extra-adrenal paraganglia (1). Although most cases exhibit an indolent clinical course, some tumors demonstrate aggressive biological behavior characterized by local invasion, recurrence, and metastasis (2). According to the 2022 World Health Organization classification, all PPGL are considered to possess metastatic potential, and malignancy is defined solely by the presence of metastasis (3). Therefore, there is a need for reliable prognostic markers that can predict the biological behavior of these tumors at the time of diagnosis.

Histopathological assessment systems such as the Pheochromocytoma of the Adrenal Gland Scaled Score (PASS) and the Grading System for Adrenal Pheochromocytoma and Paraganglioma (GAPP) have been developed for risk stratification in PPGL (4, 5). However, the accuracy of these systems in predicting metastatic potential is limited, and particularly the substantial interobserver variability of the PASS score is regarded as a major limitation (3, 6, 7). Recent molecular studies have demonstrated that hypoxia-related signaling pathways, angiogenesis, and cellular proliferation play central roles in PPGL pathogenesis and tumor progression (8, 9).

Hypoxia-inducible factor-2 alpha (HIF-2α) is a key transcription factor that is activated particularly in the pseudohypoxia subgroup and regulates angiogenesis, metabolic reprogramming, and tumor progression (10). Vascular endothelial growth factor (VEGF), one of the major effectors of HIF-mediated pathways, is among the principal regulators of tumor angiogenesis. CD105 (endoglin) is an endothelial marker that particularly reflects active neoangiogenesis and has been associated with aggressive clinical behavior and poor prognosis in various solid tumors (10, 11). Ki-67, a widely used indicator of cellular proliferation, is also included as a component of the GAPP system and is used in the prognostic assessment of PPGL (5). However, the available data regarding the relationships of these biomarkers with histopathological differentiation and tumor aggressiveness in PPGL are limited and conflicting.

In this study, the expression levels of HIF-2α, vascular endothelial growth factor (VEGF), CD105 (endoglin), Ki-67, and succinate dehydrogenase subunit B (SDHB) were evaluated immunohistochemically, and their associations with clinicopathological characteristics, PASS scores, and GAPP scores were investigated. In addition, the study aimed to determine the associations of hypoxia-, angiogenesis-, and proliferation-related biomarkers with GAPP-based histopathological differentiation status and to evaluate their associations with histopathological differentiation and histopathological risk assessment in PPGL.

## 2 Materials and methods

### 2.1 Study design and patient population

This retrospective, single-center observational study was conducted by the endocrinology and pathology departments of a tertiary training and research hospital in Türkiye. Consecutive patients who underwent surgical resection for histopathologically confirmed pheochromocytoma or intra-abdominal paraganglioma between May 2009 and October 2025 were included in the study.

Clinical records and formalin-fixed, paraffin-embedded archival tissue samples were retrospectively evaluated. Demographic data, biochemical and hormonal parameters, imaging findings, genetic analysis results, follow-up data, and macroscopic tumor characteristics (tumor size and location) were obtained from electronic medical records in accordance with ethics committee approval.

The inclusion criteria were histopathologically confirmed pheochromocytoma or intra-abdominal paraganglioma, surgical resection, and the availability of sufficient paraffin-embedded tissue material for immunohistochemical evaluation. Cases with insufficient tissue material, incomplete clinical data, or samples unsuitable for immunohistochemical examination were excluded. In addition, because the catecholamine phenotype is a component of the GAPP scoring system, cases lacking preoperative biochemical evaluation data were not included in the analysis. Head-and-neck and thoracic paragangliomas were also excluded because of differences in tumor biology and clinical behavior characteristics (3).

A total of 35 tumor foci from 31 eligible patients were examined. Of the tumor foci evaluated, 30 were classified as pheochromocytomas and 5 as intra-abdominal paragangliomas. Each anatomically distinct tumor was considered an independent tumor focus and analyzed separately. Accordingly, bilateral pheochromocytomas and synchronous pheochromocytoma and paraganglioma identified in the same patient were evaluated as separate tumor foci. For each tumor focus, one representative formalin-fixed, paraffin-embedded tissue block was selected for histopathological examination and immunohistochemical analyses.

Genetic testing was not performed systematically in all patients. Instead, genetic analyses were conducted in selected patients with clinical features suggestive of hereditary PPGL syndromes, including young age at diagnosis, bilateral or multifocal disease, and/or a family history. Genetic analyses were performed in the Department of Medical Genetics using genomic DNA extracted from peripheral EDTA-anticoagulated whole blood samples. Only germline mutations were analyzed, and somatic tumor sequencing was not performed. Germline mutation analysis was performed using next-generation sequencing and included the coding exons and exon-intron junctions of susceptibility genes. According to the clinical indication, the principal genes evaluated were rearranged during transfection (RET), Von Hippel-Lindau (VHL), neurofibromatosis type 1, and SDHx (SDHA, SDHB, SDHC, SDHD, and SDHAF2). Identified variants were interpreted according to the American College of Medical Genetics and Genomics guidelines. Genetic results were classified as pathogenic/likely pathogenic mutation detected, no pathogenic mutation detected, or genetic data unavailable.

### 2.2 Histopathological evaluation

Hematoxylin-eosin stained slides from all cases were re-evaluated by two experienced pathologists. Tumor size, location, presence of vascular invasion, and pathological T stage were recorded.

In adrenal pheochromocytoma cases, tumor aggressiveness was assessed using the PASS. In addition, the GAPP score was calculated for all pheochromocytoma and intra-abdominal paraganglioma cases. PASS and GAPP scoring was performed according to the criteria specified in the original descriptions of these systems (4, 5). The histopathological parameters constituting the PASS and GAPP scores, including tumor architecture, cellularity, necrosis, vascular and capsular invasion, mitotic activity, Ki-67 proliferation index, and other relevant criteria, were re-evaluated by two independent pathologists who were blinded to clinical outcomes. Cases with disagreement between pathologists were re-examined jointly under the microscope and consensus was reached.

According to the GAPP classification, tumors were categorized as well-differentiated (0–2 points), moderately differentiated (3–6 points), and poorly differentiated (7–10 points). For statistical analyses, well-differentiated and moderately differentiated tumors were combined into a single group and compared with poorly differentiated tumors to evaluate the association between biomarkers and differentiation status (5, 12).

### 2.3 Immunohistochemical analysis

**Figure 1:**
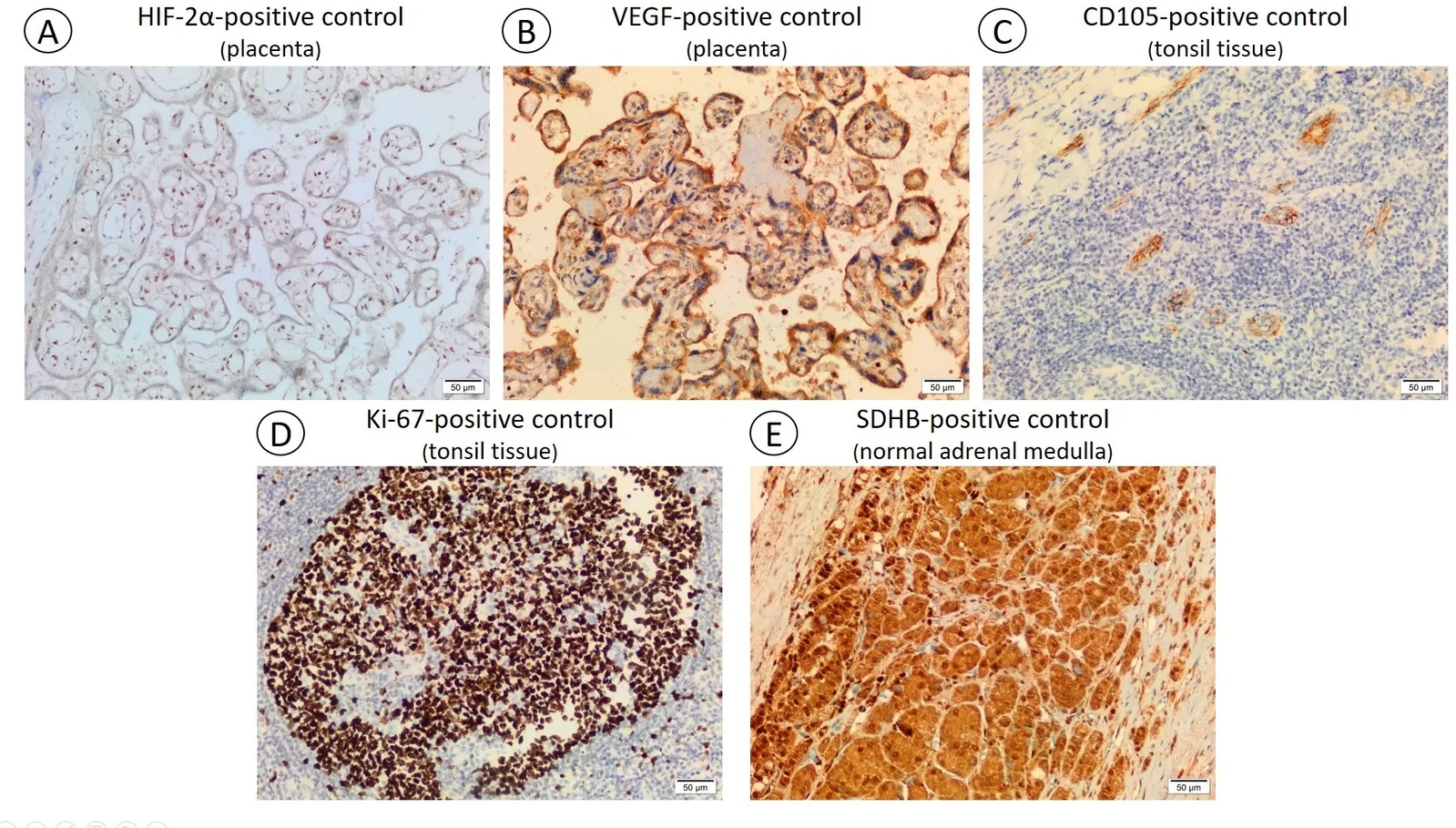
Positive control immunostaining for HIF-2α, VEGF, CD105, Ki-67, and SDHB. Representative positive control tissue sections used for immunohistochemical staining validation. **(A)** HIF-2α-positive control in human placenta, showing nuclear staining of trophoblastic cells. **(B)** VEGF-positive control in human placenta. **(C)** CD105-positive control in human tonsil, demonstrating endothelial cell staining. **(D)** Ki-67-positive control in human tonsil, showing nuclear staining in proliferating lymphoid cells. **(E)** SDHB-positive control in normal adrenal medulla, demonstrating preserved granular cytoplasmic staining. Original magnification ×200. Scale bars = 50 μm.

Formalin-fixed, paraffin-embedded tissue blocks representative of each case were selected for immunohistochemical examination. Sections prepared at a thickness of four micrometers were stained with antibodies against HIF-2α, VEGF, CD105, Ki-67, and SDHB using a fully automated immunohistochemistry staining system (Ventana BenchMark Ultra; Roche Diagnostics, Tucson, AZ, USA). The clones, manufacturers, dilution ratios, and antigen retrieval methods of the primary antibodies used are presented in **Table 1**. Appropriate positive and negative controls were processed simultaneously for all immunohistochemical stains. Representative images of the positive control tissues used for each marker are presented in **Figure 1**.

**Table 1:** Primary antibodies and immunohistochemical staining conditions.

Antibody	Clone	Manufacturer	Dilution	Antigen retrieval
HIF-2α	Polyclonal (orb1292449)	Biorbyt Ltd., Cambridge, UK	1:50	CC1
VEGF	Polyclonal (RB-9031)	Thermo Fisher Scientific (NeoMarkers), Fremont, CA, USA	1:100	CC1
CD105	SN6h	Thermo Fisher Scientific (NeoMarkers), Fremont, CA, USA	1:200	CC1
Ki-67	MIB-1	Medaysis Ltd., Liverpool, UK	1:100	CC1
SDHB	BSB-131	Bio SB Inc., Santa Barbara, CA, USA	1:100	CC1

HIF-2α, hypoxia-inducible factor-2 alpha; VEGF, vascular endothelial growth factor; SDHB, succinate dehydrogenase subunit B; CC1, cell conditioning 1.

In PPGL, hemorrhage, cystic degeneration, and other secondary changes are known to affect vascular architecture and immunohistochemical evaluation results. Therefore, all immunohistochemical analyses were performed in areas representing well-preserved and viable tumor tissue (13).

HIF-2α expression was evaluated using the semi-quantitative H-score method. Staining intensity was graded as 0 (no staining), 1+ (weak), 2+ (moderate), and 3+ (strong). The H-score was calculated as the sum of the products of the percentage of positive cells in each intensity category and the corresponding intensity score, yielding values ranging from 0 to 300. H-score assessment was performed manually under conventional light microscopy by two independent pathologists blinded to the clinical data. Digital image analysis was not used.

VEGF expression was evaluated using the immunoreactivity score (IRS) method. Staining intensity was scored as 0 (no staining), 1 (weak), 2 (moderate), and 3 (strong). The proportion of positive tumor cells was graded as 0 (0%), 1 (<10%), 2 (10–50%), 3 (51–80%), and 4 (>80%). The IRS was calculated by multiplying the staining intensity score by the positive cell proportion score, resulting in values ranging from 0 to 12. Higher IRS values represented higher VEGF expression.

CD105 expression was evaluated as an indicator of tumor angiogenesis using the microvessel density (MVD) method. First, areas with the highest vascular density (“hot spots”) were identified under low magnification (×40–100). Subsequently, under high magnification (×400), CD105-positive endothelial cells or endothelial cell clusters clearly separated from adjacent structures, regardless of the presence of a lumen, were counted as a single microvascular structure. The mean count from three separate hot-spot areas for each case was recorded as the CD105-MVD value.

The Ki-67 proliferation index was calculated by evaluating at least 500 tumor cells in areas showing the highest proliferative activity and was reported as the percentage of cells exhibiting positive nuclear staining.

SDHB immunohistochemical expression was evaluated in conjunction with internal positive controls. The presence of distinct granular cytoplasmic staining in tumor cells was considered retained expression. Complete loss of staining or the presence of only weak diffuse cytoplasmic staining in tumor cells, provided that positive staining was preserved in internal control cells, was considered loss of SDHB expression.

All immunohistochemical evaluations were performed independently by two pathologists blinded to the clinical data, and cases with disagreement were resolved through joint review. Interobserver agreement was assessed using the intraclass correlation coefficient (ICC) for continuous variables and Cohen’s kappa coefficient for SDHB expression.

**Figure 2:**
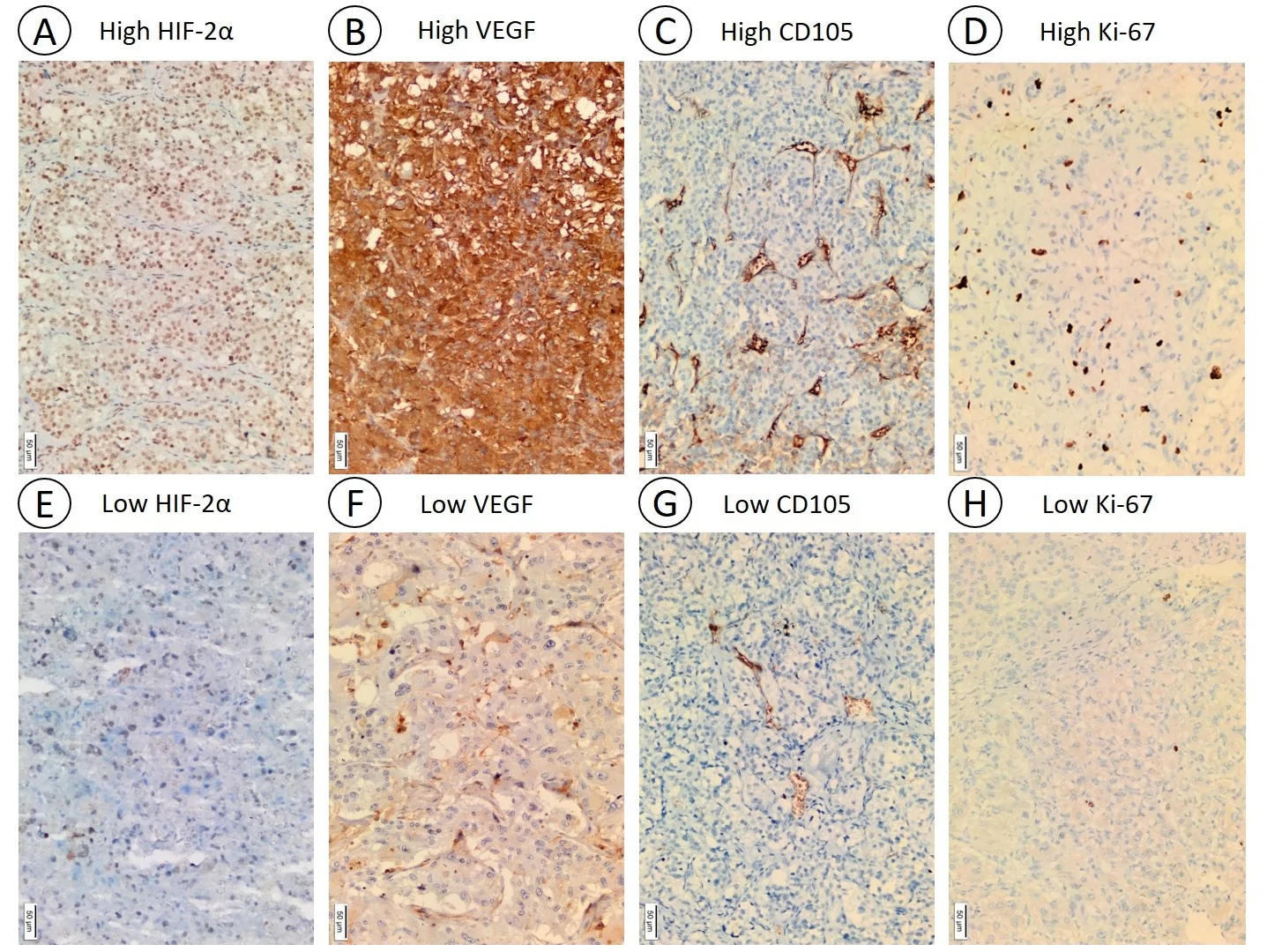
Representative immunohistochemical expression patterns of HIF-2α, VEGF, CD105, and Ki-67 in pheochromocytomas and paragangliomas (PPGLs). Representative images showing the highest and lowest expression levels of the investigated immunohistochemical markers. Panels **(A–D)** demonstrate the highest expression levels of HIF-2α **(A)**, VEGF **(B)**, CD105 **(C)**, and Ki-67 **(D)**, respectively. Panels E–H demonstrate the lowest expression levels of HIF-2α **(E)**, VEGF **(F)**, CD105 **(G)**, and Ki-67 **(H)**, respectively. All images were obtained at ×200 magnification. Scale bar = 50 μm.

**Figure 3:**
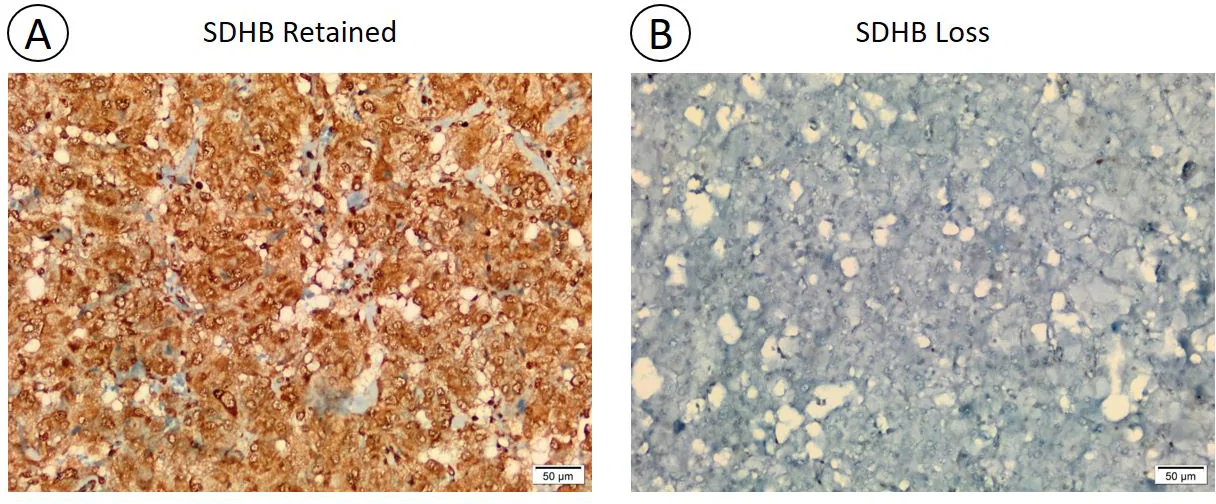
Representative SDHB immunohistochemical staining patterns in PPGLs. Representative images demonstrating retained SDHB expression **(A)** and loss of SDHB expression **(B)** in PPGL specimens. All images were obtained at ×200 magnification. Scale bar = 50 μm.

Representative immunohistochemical staining patterns from cases demonstrating the highest and lowest expression levels of HIF-2α, VEGF, CD105, and Ki-67 are shown in **Figure 2**. Representative SDHB immunohistochemical staining patterns demonstrating retained and lost SDHB expression are presented in **Figure 3**.

### 2.4 Statistical analysis

Statistical analyses were performed using R software (Version 4.3.2, R Foundation for Statistical Computing, Vienna, Austria). Continuous variables were presented as mean ± standard deviation or median (interquartile range), and categorical variables as number and percentage. Welch’s t-test was used for comparisons of continuous variables between groups, and Fisher’s exact test was used for categorical variables. Interobserver agreement for continuous immunohistochemical variables was evaluated using ICC based on a two-way mixed-effects, absolute-agreement, single-measurement model. For SDHB expression, Cohen’s kappa (κ) coefficient was calculated. ICC values were interpreted according to the recommendations of Koo and Li (14).

Associations between histopathological scores and immunohistochemical markers were evaluated using Spearman correlation analysis. To identify factors associated with the GAPP score, univariate linear regression analyses were first performed. Although the GAPP score is an ordinal grading system, it was treated as a continuous variable in the regression analyses to evaluate factors associated with changes in this score. Ordinal regression analysis was not performed because of the limited sample size and the small number of cases in some GAPP categories. Variables found to be significant in the univariate analyses were subsequently included in a multivariable linear regression model to investigate independent determinants. Statistical significance was defined as p<0.05.

## 3 Results

### 3.1 Demographic and clinical characteristics of patients

A total of 31 patients with PPGL were included in the study. The mean age of the patients was 49.39 ± 15.26 years, and 54.8% were male. Histopathological and immunohistochemical evaluations were performed on a total of 35 tumor foci from these patients. Of the tumor foci examined, 30 were classified as pheochromocytomas and 5 as intra-abdominal paragangliomas. On a patient basis, 26 patients had isolated pheochromocytoma, 4 had isolated paraganglioma, and 1 had concomitant pheochromocytoma and paraganglioma. Bilateral pheochromocytomas were present in 3 patients.

Genetic evaluation was available for 23 of the 31 patients. Pathogenic mutations associated with PPGL were identified in 5 patients (16.1%) of the entire cohort, including 3 mutations in the VHL gene and 2 in the RET gene. No mutation was detected in 18 patients, while genetic data were unavailable for 8 patients. The mean clinical follow-up duration was 50.26 ± 45.51 months. During follow-up, distant metastasis was identified in 3 patients (9.7%). The demographic, clinical, genetic, and catecholamine secretion phenotype data of the study cohort are summarized in **Table 2**.

**Table 2:** Demographic and clinical characteristics of the study cohort.

Variable	Total cohort (n = 31)^1^
Age (years)	49.39 ± 15.26
Sex	
- Male	17 (54.84)
- Female	14 (45.16)
Diagnosis	
- Pheochromocytoma	26 (83.87)
- Isolated paraganglioma	4 (12.90)
- Combined (PPGL)	1 (3.23)
**Bilateral disease**	3 (9.68)
Genetic mutation status^2^	
- Positive	5 (16.13)
- Negative	18(58.06)
- Unknown	8(25.81)
Mutation type^3^	
- RET	2 (40.00)
- VHL	3 (60.00)
Follow-up duration (months)	50.26 ± 45.51
Distant metastasis	
- Negative	24 (77.42)
- Positive	3 (9.68)
- Unknown	4 (12.90)
Catecholamine secretion phenotype^4^	
- Predominantly normetanephrine	13 (41.94)
- Predominantly metanephrine	1(3.23)
- Combined (metanephrine + normetanephrine)	11(35.48)
- Predominantly dopamine	2(6.45)
- Biochemically negative (normal metabolites)	4(12.90)

^1^Data are presented as mean ± standard deviation or n (%).

^2^Genetic testing was not routinely performed in the entire cohort and was limited to selected patients with clinical suspicion of hereditary disease. Genetic mutation status was classified as positive, negative, or unknown according to the availability and results of genetic testing.

^3^Mutation subtype analysis was performed only among mutation-positive patients (n = 5).

^4^The catecholamine secretion phenotype was determined according to the predominant elevation in plasma or 24-hour urinary catecholamine metabolites. Because the biochemical evaluation was performed using plasma metabolite measurements in some patients and 24-hour urinary metabolite measurements in others, absolute metabolite levels were not directly comparable across patients and were therefore not presented for the cohort as a whole.

PPGL, pheochromocytoma/paraganglioma; RET, rearranged during transfection proto-oncogene; VHL, Von Hippel-Lindau.

### 3.2 Histopathological characteristics and immunohistochemical marker profiles

The histopathological and immunohistochemical characteristics of all 35 tumor foci were evaluated. Of the tumors, 11(31.4%) were classified as poorly differentiated, while vascular invasion and loss of SDHB expression were identified in7(20.0%) and 4(11.4%) of cases, respectively. Detailed histopathological and immunohistochemical findings are presented in **Table 3**.

**Table 3:** Tumor characteristics, histopathological findings, and immunohistochemical marker profiles of the evaluated tumor foci.

Variable	Tumor Foci (n=35)^1^
Tumor diameter (mm)	58.74 ± 31.12
Pathological T stage	
pT1	16 (45.71)
pT2	19 (54.29)
Tumor localization	
Abdominal	5 (14.29)
Left adrenal	15 (42.86)
Right adrenal	15 (42.86)
PASS score	6.07 ± 3.22
GAPP score	5.60 ± 2.00
GAPP group*	
Well differentiated	4 (11.43)
Moderately differentiated	20 (57.14)
Poorly differentiated	11 (31.43)
Vascular invasion	7 (20.00)
SDHB status	
Retained	31 (88.57)
Loss	4 (11.43)
Immunohistochemical marker profiles	
HIF-2α H-score	87.14 ± 78.20
VEGF IRS score	4.29 ± 3.29
CD105 MVD score	21.46 ± 19.82
Ki-67 index (%)	2.00 (1.00–3.00)

^1^Data are presented as mean ± standard deviation, median (Q1–Q3), or n (%).

*GAPP groups were classified as well differentiated (0–2), moderately differentiated (3–6), and poorly differentiated (7–10) (5, 12).

PASS, Pheochromocytoma of the Adrenal Gland Scaled Score;

GAPP, Grading system for Adrenal Pheochromocytoma and Paraganglioma; SDHB, succinate dehydrogenase subunit B; HIF-2α, hypoxia-inducible factor-2 alpha; VEGF, vascular endothelial growth factor; IRS, immunoreactivity score; MVD, microvessel density.

Excellent interobserver agreement was observed between the two pathologists for the assessment of HIF-2α, VEGF IRS score, CD105 MVD score, and Ki-67 (all ICC values >0.90). The highest agreement was observed for HIF-2α assessment (ICC = 0.979), whereas the lowest agreement was observed for VEGF IRS score assessment (ICC = 0.931). Complete agreement was achieved between the two pathologists in the assessment of SDHB expression (Cohen’s κ = 1.00; observed agreement = 100%). Detailed interobserver agreement results are presented in **Supplementary Table 1**.

### 3.3 Correlations between histopathological risk scores and immunohistochemical markers

**Figure 4:**
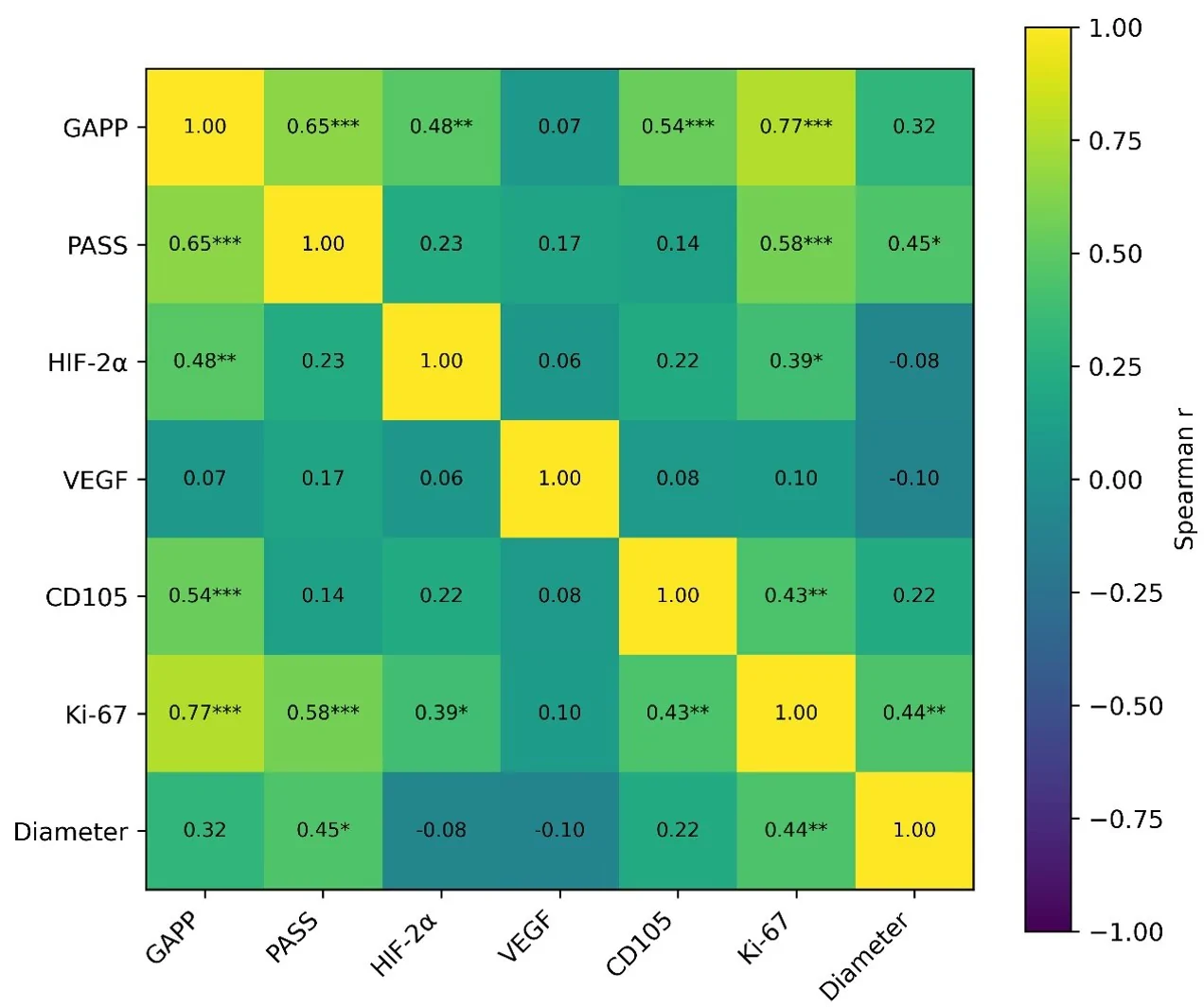
Heatmap showing Spearman correlations between histopathological scores and immunohistochemical biomarkers in PPGL. The heatmap illustrates the correlations of GAPP and PASS scores with HIF-2α, VEGF, CD105, Ki-67 proliferation index, and tumor size. Each cell represents the Spearman correlation coefficient (r) for the corresponding variable pair. Color intensity indicates the direction and strength of the correlation. Statistically significant correlations are denoted by *, **, and *** (*p < 0.05, **p < 0.01, ***p < 0.001).

Spearman correlation analysis demonstrated that the GAPP score was significantly positively correlated with HIF-2α, CD105, and Ki-67. The PASS score was positively correlated with Ki-67 and tumor size. In addition, the Ki-67 proliferation index exhibited significant correlations with HIF-2α, CD105, and tumor size. In contrast, no significant associations were observed between VEGF expression and either histopathological scores or other immunohistochemical markers. Detailed results of the correlation analysis are shown in **Figure 4**.

### 3.4 Clinicopathological and immunohistochemical features according to GAPP differentiation status

**Figure 5:**
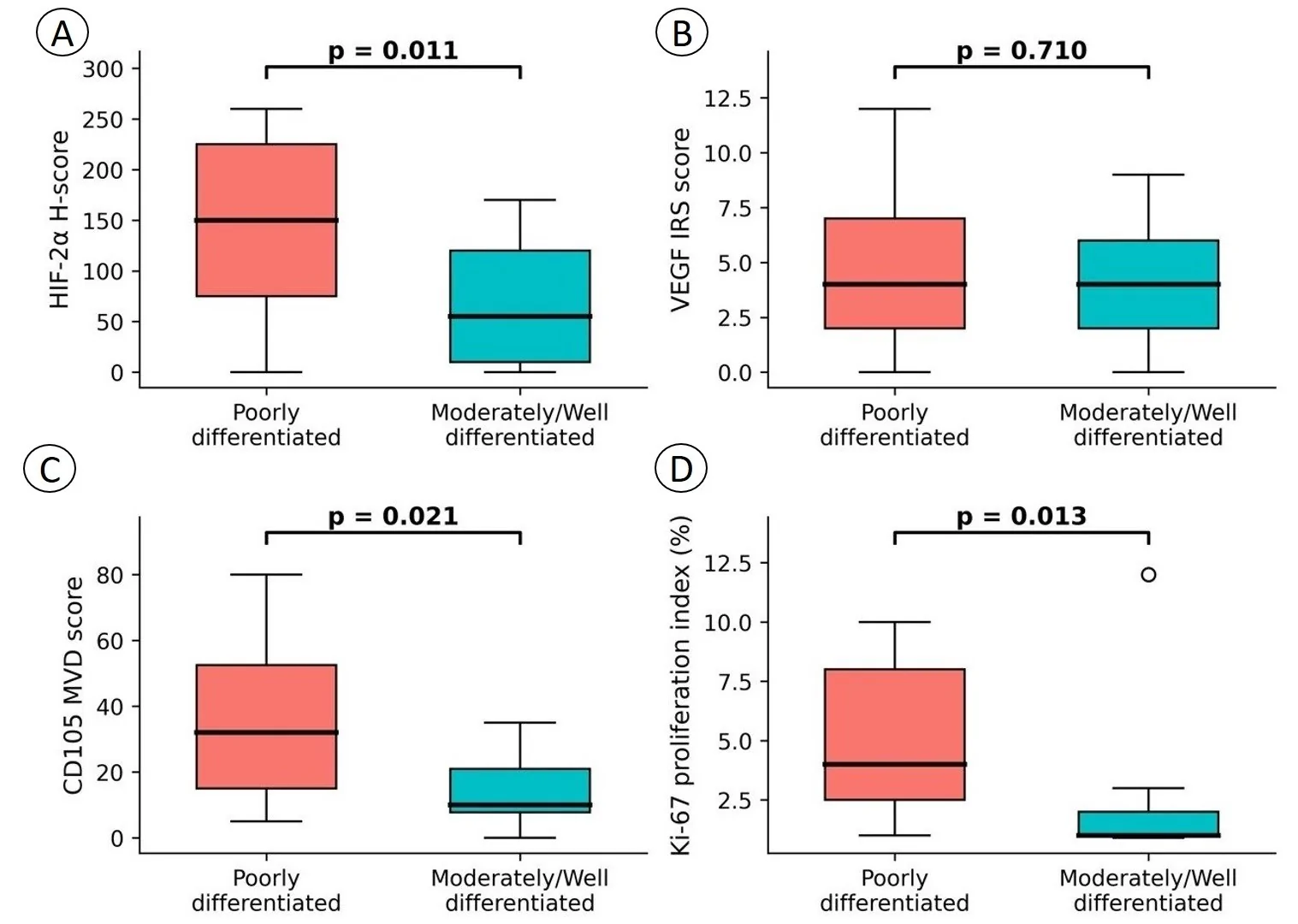
Immunohistochemical biomarker expression according to GAPP differentiation status. Box plots showing the distribution of HIF-2α H-score **(A)**, VEGF IRS score **(B)**, CD105 microvessel density (MVD) score **(C)**, and Ki-67 proliferation index **(D)** according to GAPP differentiation status. HIF-2α expression, CD105 MVD score, and Ki-67 proliferation index were significantly higher in poorly differentiated tumors compared with moderately/well-differentiated tumors. In contrast, no significant difference was observed between the groups in terms of VEGF IRS scores. P-values are shown above each comparison.

Poorly differentiated tumors (GAPP 7–10) were compared with well- and moderately differentiated tumors (GAPP 0–6). The frequencies of bilateral disease and vascular invasion were significantly higher in the poorly differentiated group. In addition, PASS scores were significantly increased in this group. Immunohistochemical analyses demonstrated that the HIF-2α H-score, CD105 MVD score, and Ki-67 proliferation index were significantly higher in poorly differentiated tumors. In contrast, no significant difference was observed between the groups in terms of VEGF IRS scores. Although tumor size was greater in the poorly differentiated group, this difference did not reach statistical significance. Comparisons of clinicopathological and immunohistochemical characteristics according to GAPP differentiation status are presented in **Table 4**. Box plots showing the distribution of immunohistochemical markers according to GAPP differentiation status are presented in **Figure 5**.

**Table 4:** Clinicopathological and immunohistochemical features according to GAPP differentiation status.

Variable	Poorly differentiated (n=11)^1^	Moderately/well differentiated (n=24)^1^	p^2^ value
GAPP score	7.73 ± 1.19	4.63 ± 1.47	<0.001
PASS score	8.55 ± 3.14	4.63 ± 2.29	0.002
Tumor diameter (mm)	71.27 ± 22.02	53.00 ± 33.34	0.065
Bilateral disease, n (%)	5 (45.5)	2 (8.3)	0.021
Vascular invasion, n (%)	6 (54.5)	1 (4.2)	0.002
HIF-2α H-score	145.45 ± 88.70	60.42 ± 56.99	0.011
VEGF IRS score	4.64 ± 3.91	4.13 ± 3.04	0.70
CD105 MVD score	36.82 ± 26.97	14.42 ± 10.00	0.021
Ki-67 index (%)	5.09 ± 3.45	1.85 ± 2.24	0.013

**^1^**Data are presented as mean ± standard deviation or n (%).

**^2^**p values were calculated using Welch’s t-test or Fisher’s exact test, as appropriate.

PASS, Pheochromocytoma of the Adrenal Gland Scaled Score; GAPP, Grading System for Adrenal Pheochromocytoma and Paraganglioma; HIF-2α, hypoxia-inducible factor-2 alpha; VEGF, vascular endothelial growth factor; IRS, immunoreactivity score; MVD, microvessel density.

### 3.5 Associations of immunohistochemical markers with genetic mutation status, SDHB expression, and vascular invasion

Immunohistochemical marker profiles were compared according to available genetic analysis results, SDHB expression status, and the presence of vascular invasion. Evaluation of 27 tumor foci from patients with available genetic analysis results demonstrated that VEGF IRS scores were significantly higher in tumors from patients with pathogenic mutations. Evaluation of 27 tumor foci from patients with available genetic analysis results demonstrated that VEGF IRS scores were significantly higher in the group with pathogenic mutations.

In contrast, no significant differences were observed in GAPP score, HIF-2α H-score, CD105 MVD score, or Ki-67 proliferation index.

Tumors with loss of SDHB expression showed higher GAPP scores however, these differences did not reach statistical significance. Similarly, although GAPP scores were significantly higher in tumors positive for vascular invasion, no significant differences were observed in HIF-2α, CD105, or Ki-67 levels.

Comparisons of immunohistochemical marker profiles according to clinicopathological subgroups are presented in **Supplementary Table 2**.

### 3.6 Univariate analysis of factors associated with GAPP score

In univariate linear regression analysis, the presence of bilateral disease, higher HIF-2α H-score, higher CD105 MVD score, and loss of SDHB expression were significantly associated with higher GAPP scores. In contrast, no significant associations were found between GAPP score and age, sex, tumor location, tumor size, presence of a genetic mutation, or VEGF IRS score. The results of univariate linear regression analyses of clinicopathological and immunohistochemical variables associated with the GAPP score are presented in **Table 5**.

**Table 5:** Univariate linear regression analysis for GAPP score.

Variable	β	95% CI	P value
Age	-0.04	-0.08, 0.00	0.071
Female sex	0.05	-1.4, 1.4	0.950
Bilateral disease (present)	2.5	0.95, 4.00	0.002
Left adrenal localization	1.9	-0.12, 4.0	0.170
Right adrenal localization	1.3	-0.72, 3.4	0.170
Tumor diameter	0.01	-0.01, 0.04	0.190
Positive genetic mutation	1.0	-0.68, 2.7	0.460
HIF-2α H-score	0.01	0.00, 0.02	0.003
VEGF IRS score	0.07	-0.15, 0.28	0.540
CD105 MVD score	0.05	0.02, 0.09	<0.001
SDHB loss	2.40	0.41, 4.40	0.020

Data are presented as regression coefficients (β), 95% confidence intervals (CI), and p-values.

CI, confidence interval; SDHB, succinate dehydrogenase subunit B; HIF-2α, hypoxia-inducible factor-2 alpha; VEGF, vascular endothelial growth factor; IRS, immunoreactivity score; MVD, microvessel density.

### 3.7 Independent predictors of GAPP score

Variables that were significantly associated with the GAPP score in the univariate analysis were included in a multivariable linear regression model. In the multivariable analysis, only the CD105 MVD score remained independently associated with the GAPP score. HIF-2α H-score and SDHB expression status did not retain statistical significance in the multivariable model. The final model explained 41.4% of the variance in the GAPP score (R² = 0.414). The results of the multivariable linear regression analysis are presented in **Table 6**.

**Table 6:** Multivariable linear regression analysis for GAPP score.

Variable	β	95% CI	P value
HIF-2α H-score	0.01	0.00, 0.01	0.091
CD105 MVD score	0.04	0.00, 0.07	0.026
SDHB loss	1.3	0.49, 3.2	0.140

Model R² = 0.414.

CI, confidence interval; SDHB, succinate dehydrogenase subunit B; HIF-2α, hypoxia-inducible factor-2 alpha; MVD, microvessel density.

## 4 Discussion

Elucidating the relationship between histopathological risk classification systems and molecular biomarkers in PPGL is important for improving our understanding of tumor biology and histopathological risk assessment. This study evaluated the expression of SDHB, HIF-2α, VEGF, CD105, and Ki-67 in 35 PPGL tumor foci from 31 patients and investigated the associations of these biomarkers with histopathological risk indicators.

Our findings demonstrated that HIF-2α, CD105, and Ki-67 expression levels were significantly increased in poorly differentiated tumors according to the GAPP classification and were positively correlated with the GAPP score. In contrast, the PASS score showed a significant association only with Ki-67, whereas no significant associations were observed with HIF-2α, CD105, or VEGF expression. Furthermore, VEGF expression was not associated with either tumor differentiation or histopathological risk classification systems. In the multivariable analysis, only CD105 remained independently associated with the GAPP score, suggesting that CD105 expression may reflect biological processes related to histopathological differentiation in PPGL. Taken together, our findings suggest that immunohistochemical biomarkers associated with hypoxia, angiogenesis, and proliferation are related to histopathological differentiation and GAPP-based histopathological risk classification in PPGL. However, their associations with clinical aggressiveness and prognosis should be confirmed in larger studies with long-term clinical outcome data.

HIF-2α is one of the key regulators of pseudohypoxia pathways that play a central role in PPGL biology and controls the expression of numerous target genes associated with tumor progression (15, 16). Previous studies have shown that increased HIF-2α expression may be associated with higher histopathological risk, poor differentiation, and metastatic potential (10, 14). Consistent with these observations, our findings demonstrated that HIF-2α expression was higher in poorly differentiated tumors and positively correlated with the GAPP score. In contrast, no significant association was observed with the PASS score. Furthermore, the lack of an independent association of HIF-2α in the multivariable analysis suggests that its prognostic impact may be influenced by other molecular and histopathological factors. Given the limited number of studies evaluating the relationship between HIF-2α expression and GAPP-based histopathological risk stratification, our findings provide additional evidence supporting the association of HIF-2α with tumor differentiation and histopathological risk in PPGL. The association of HIF-2α with poor differentiation and higher GAPP scores further supports the importance of pseudohypoxia pathways in PPGL biology. However, its independent prognostic value should be confirmed in larger cohorts and through long-term clinical outcome data.

One of the most noteworthy findings of our study concerns CD105, which is recognized as an angiogenesis marker expressed in actively proliferating endothelial cells and reflecting tumor-associated neovascularization (17). However, accumulating evidence in recent years has shown that CD105 is not merely an indicator of tumor angiogenesis but also participates in biological mechanisms related to tumor microenvironment remodeling, cellular differentiation processes, and treatment resistance (18). Tumor microvessel density assessed by CD105 has been reported to be associated with tumor aggressiveness, metastatic potential, and adverse clinical outcomes in many solid tumors, particularly breast, colorectal, prostate, and head-and-neck cancers (11, 18–21). In our previous study on papillary thyroid carcinoma, we also demonstrated that CD105 expression was associated with lymph node metastasis, distant metastasis, and high-risk clinicopathological features (22). From this perspective, the present findings suggest that CD105 may reflect biological processes associated with histopathological differentiation across endocrine tumors.

Consistent with these observations, our findings demonstrated that the CD105 MVD score was significantly increased in poorly differentiated tumors, showed a strong positive correlation with the GAPP score, and was the only variable independently associated with GAPP in the multivariable analysis. Furthermore, the positive correlation observed between CD105 and Ki-67 suggests that increased neovascularization and proliferative activity may progress concurrently. The finding that GAPP scores were significantly higher in cases with vascular invasion, together with the observation that HIF-2α and CD105 levels were approximately twofold higher in tumors with vascular invasion than in those without vascular invasion, although not reaching statistical significance, suggests that hypoxia-related angiogenic processes may contribute to invasive tumor behavior in PPGL. On the other hand, the absence of a significant correlation between HIF-2α and CD105 suggests that angiogenesis in PPGL is a complex process regulated not only by hypoxia signaling but also by interactions among genetic background, the tumor microenvironment, and various proangiogenic mechanisms.

The number of studies investigating the prognostic value of CD105 in PPGL remains very limited. In the few available studies, microvessel density assessed by CD105 was not significantly associated with malignant potential or PASS score (13, 23). Similarly, in our study, no significant correlation was observed between the CD105 MVD score and PASS. In contrast, the strong association between CD105 and the GAPP score is noteworthy. Whereas PASS is based predominantly on histomorphological features, GAPP provides a more comprehensive biological risk assessment incorporating differentiation grade, catecholamine phenotype, and the Ki-67 proliferation index. Supporting this, previous studies have reported that higher GAPP scores are associated with more aggressive PPGL behavior, whereas the PASS score has limited utility in predicting metastatic potential (7). In this context, the strong association observed between CD105 and the GAPP score suggests that CD105 expression may reflect biological processes related to histopathological differentiation in PPGL and may contribute to a better understanding of tumor biology.

Although VEGF is considered one of the principal regulators of tumor angiogenesis (24), no significant association was observed in our study between VEGF expression and GAPP score, PASS score, or tumor differentiation. Similarly, the literature contains conflicting findings regarding the prognostic significance of VEGF. While some studies have linked VEGF-mediated angiogenesis with malignant behavior, others have failed to demonstrate a significant association between VEGF expression or microvessel density and metastatic potential (13, 25). Nevertheless, the observation of higher VEGF expression in mutation-positive cases, a substantial proportion of which consisted of VHL-mutant tumors, is noteworthy. Loss of VHL protein function prevents the degradation of HIF-2α, leading to activation of pseudohypoxic signaling and subsequent upregulation of numerous angiogenic target genes, particularly VEGF (26). Indeed, Pollard et al. demonstrated that HIF-2α and VEGF expression levels were significantly higher in VHL-mutant pheochromocytomas than in SDHB/SDHD-associated tumors (27). Nevertheless, VEGF expression may not be a marker that directly reflects active neovascularization or histopathological differentiation. VEGF expression can be influenced by multiple factors, including the underlying molecular subgroup, the tumor microenvironment, and the dynamic regulation of hypoxia signaling (9, 10, 23). In contrast, CD105 is a marker that more directly reflects active endothelial proliferation and neovascularization. This biological difference may explain why CD105 showed a significant association with GAPP-based histopathological differentiation in our study, whereas VEGF did not. These findings suggest that VEGF expression may reflect molecular activation of the VHL-HIF-2α axis rather than histopathological aggressiveness.

Ki-67 was the only immunohistochemical marker that showed a strong positive correlation with both GAPP and PASS scores and a significant association with the PASS score. However, the association between Ki-67 and the GAPP score should be interpreted with caution because Ki-67 is an integral component of the GAPP scoring system. Therefore, the observed association is partly attributable to the mathematical structure of the scoring system and should not be considered independent biological evidence (5). In contrast, because Ki-67 is not included in the PASS scoring system, the association observed with PASS is not affected by the same methodological limitation and may reflect a relationship between proliferative activity and the histopathological features assessed by PASS (5, 28, 29). Furthermore, the positive correlations of Ki-67 with tumor size, HIF-2α, and CD105 suggest that proliferative activity may be associated with hypoxia and tumor-associated angiogenesis. Taken together, these findings indicate that immunohistochemical biomarkers are associated with the histopathological features evaluated by PASS and GAPP but do not directly demonstrate an association with clinical outcomes or prognosis (26, 27, 30).

Loss of SDHB expression is one of the most important molecular markers associated with poor prognosis and metastatic potential in PPGL (3, 31). Accordingly, the modified GAPP model, which incorporates SDHB status, was developed, and it has been proposed that SDHB loss may provide additional prognostic value in risk stratification (12). In our study, poor differentiation was observed in three of the four cases exhibiting loss of SDHB expression, and SDHB loss was associated with higher GAPP scores in univariate regression analysis. In addition, HIF-2α and CD105 levels tended to be higher in cases with SDHB loss than in tumors with retained SDHB expression. Although these differences did not reach statistical significance, our findings suggest that SDHB loss may be associated with increased histopathological risk as well as elevated markers of pseudohypoxia and tumor-associated angiogenesis. These observations are consistent with previous studies demonstrating that pseudohypoxia pathways resulting from SDHB inactivation shape PPGL biology by triggering HIF-mediated transcriptional activation and angiogenic processes (8, 30, 32). Nevertheless, the identification of loss of SDHB expression in only four tumors limited the statistical power, and these findings should therefore be interpreted with caution. There is a need for validation through larger studies.

One of the noteworthy findings of our study was that, although both HIF-2α and CD105 expression were associated with poorer histopathological differentiation, no significant correlation was observed between these two biomarkers. Although this finding may initially appear unexpected, it may be explained by the current molecular classification of PPGL, which categorizes tumors into the pseudohypoxia (Cluster 1), kinase signaling (Cluster 2), and Wnt-altered subgroups, with the pseudohypoxic subgroup exhibiting considerable biological heterogeneity (8–10). Supporting this, although both Cluster 1A tumors (harboring tricarboxylic acid cycle-related mutations such as SDHx, FH, and MDH2) and Cluster 1B tumors (harboring mutations such as VHL and EPAS1) belong to the pseudohypoxic subgroup, they differ substantially in HIF signaling activation, metabolic reprogramming, and the regulation of angiogenesis (8, 10). Furthermore, immunohistochemical HIF-2α expression reflects only protein expression, whereas the functional transcriptional activity of HIF-2α depends on additional regulatory mechanisms, including nuclear translocation, interaction with cofactors, and activation of downstream target genes. Therefore, the immunohistochemical expression level may not always accurately reflect the functional hypoxic response and angiogenic activity within the tumor microenvironment (10). This may explain why CD105 expression remained independently associated with the GAPP score, whereas HIF-2α did not emerge as an independent predictor in the multivariable analysis. Moreover, the absence of a significant correlation between HIF-2α and CD105 suggests that angiogenesis in PPGL cannot be explained solely by HIF-2α-mediated pseudohypoxia signaling and that the tumor microenvironment, stromal interactions, and other proangiogenic mechanisms may also contribute to this process (9). Although comprehensive molecular subgroup analysis was not performed in the current study, our findings suggest that CD105 may not merely reflect HIF-2α activation but may instead represent processes associated with functional angiogenic remodeling in PPGL. Nevertheless, these interpretations should be considered with caution because of the limited molecular characterization in our study. Further studies in larger, molecularly characterized, multicenter cohorts, particularly including Cluster 1A and Cluster 1B tumors, are required to validate these observations.

Although this study focused on tissue-based biomarkers, functional imaging biomarkers have also gained increasing importance in the prognostic assessment of PPGL in recent years (2, 33). A recent study demonstrated that volumetric metabolic parameters derived from ^18F-FDG PET/CT, particularly metabolic tumor volume and total lesion glycolysis, were significantly associated with overall survival after ^131I-MIBG therapy in patients with unresectable PPGL and provided stronger prognostic information than SUVmax, one of the conventional PET parameters (33). These findings suggest that imaging biomarkers may reflect tumor metabolic burden and biological behavior beyond that captured by histopathological evaluation. Although functional imaging parameters were not assessed in the present study, combined evaluation of tissue biomarkers reflecting hypoxia, angiogenesis, and proliferation, such as HIF-2α, CD105, and Ki-67, together with functional imaging biomarkers such as metabolic tumor volume and total lesion glycolysis, may contribute to a more comprehensive understanding of PPGL biology and facilitate the development of more accurate risk stratification models in the future. Therefore, there is a need for prospective multicenter studies integrating molecular pathology, functional imaging, and long-term clinical outcomes.

This study has several limitations. Its retrospective, single-center design and relatively small sample size may limit the generalizability of the findings. In addition, the inclusion of patients over an approximately 16-year period (2009–2025) may have introduced cohort heterogeneity because of temporal changes in diagnostic approaches, genetic testing strategies, imaging modalities, and immunohistochemical practices. To create a more homogeneous cohort, only abdominal PPGLs were included, which limits the generalizability of the findings to paragangliomas arising at other anatomical sites. Furthermore, although pheochromocytomas constituted the majority of the cohort, intra-abdominal paragangliomas, which are generally associated with more aggressive clinical behavior, were represented by only a limited number of cases. In addition, because 35 tumor foci were obtained from 31 patients, the observations were not completely independent. Failure to account for the potential correlation between multiple tumor foci from the same patient in the statistical analyses may have resulted in overestimation of the effective sample size and may have affected the estimates of standard errors and statistical significance. Therefore, the findings of the correlation and multivariable analyses, in particular, should be interpreted with caution. Because the number of patients with multiple tumor foci was very small, mixed-effects models accounting for patient-level clustering were not applied. Due to the presence of only three metastatic cases, direct associations between the investigated biomarkers and metastatic behavior, recurrence, or other clinical outcomes could not be evaluated. Accordingly, our study assessed tumor aggressiveness using histopathological risk classification systems rather than clinical outcomes. Furthermore, the catecholamine phenotype component of the GAPP score was determined using plasma or 24-hour urinary metanephrine measurements, depending on data availability. Although the phenotype was determined according to the predominant metabolite elevation rather than direct comparison of absolute concentrations, the use of two different specimen types may have introduced measurement heterogeneity into the GAPP classification. Moreover, because Ki-67 is an integral component of the GAPP scoring system, part of the observed association between Ki-67 and the GAPP score may be attributable to the mathematical structure of the scoring system rather than a completely independent biological relationship. Therefore, this finding should be interpreted cautiously as a methodological limitation. In addition, the limited number of tumors with poor prognostic features, particularly those harboring SDHB mutations or showing loss of SDHB expression, restricted reliable evaluation of the prognostic significance of the investigated immunohistochemical biomarkers in this high-risk subgroup. The availability of genetic data only for selected cases and the evaluation of a limited number of susceptibility genes also precluded a comprehensive assessment of the relationships between molecular subgroups and immunohistochemical biomarkers. Finally, because recurrence and metastasis in PPGL may occur years after diagnosis, larger multicenter studies with longer follow-up are required to determine the long-term prognostic value of these biomarkers.

In conclusion, our findings suggest that biomarkers associated with hypoxia, angiogenesis, and cellular proliferation are related to histopathological risk indicators in PPGL. HIF-2α, CD105, and Ki-67 expression levels were increased in tumors with higher histopathological risk scores and were positively correlated with the GAPP score. Among the biomarkers evaluated, only CD105 remained independently associated with the GAPP score, suggesting that CD105 expression may reflect biological processes related to histopathological differentiation in PPGL. In contrast, VEGF expression was not significantly associated with histopathological risk indicators. Overall, our findings indicate that the immunohistochemical biomarkers evaluated are associated with histopathological differentiation and GAPP-based risk classification. However, because our study evaluated associations with histopathological risk classification systems rather than clinical outcomes, larger studies incorporating comprehensive molecular characterization and long-term clinical follow-up are needed to determine whether these findings are associated with clinically meaningful outcomes.

## Data Availability

The dataset is publicly released and accessible in Zenodo (DOI: 10.5281/zenodo.20785485; direct record: https://zenodo.org/records/20785485).

## References

[1] Tanabe A Katabami T Hashimoto S Izawa S Ichijo T Otsuki M . Japan Endocrine Society Clinical Practice Guideline for the diagnosis and management of pheochromocytoma and paraganglioma 2025. Endocr J. (2026) 73:115–57. doi: 10.1507/endocrj.EJ25-0165 41083371 PMC12819074

[2] Nölting S Bechmann N Taieb D Beuschlein F Fassnacht M Kroiss M . Personalized management of pheochromocytoma and paraganglioma. Endocr Rev. (2022) 43:199–239. doi: 10.1210/endrev/bnab019 34147030 PMC8905338

[3] Mete O Asa SL Gill AJ Kimura N de Krijger RR Tischler A . Overview of the 2022 WHO classification of paragangliomas and pheochromocytomas. Endocr Pathol. (2022) 33:90–114. doi: 10.1007/s12022-022-09704-6 35285002

[4] Thompson LD . Pheochromocytoma of the adrenal gland scaled score (PASS) to separate benign from Malignant neoplasms: a clinicopathologic and immunophenotypic study of 100 cases. Am J Surg Pathol. (2002) 26:551–66. doi: 10.1097/00000478-200205000-00002 11979086

[5] Kimura N Takayanagi R Takizawa N Itagaki E Katabami T Kakoi N . Pathological grading for predicting metastasis in phaeochromocytoma and paraganglioma. Endocr Relat Cancer. (2014) 21:405–14. doi: 10.1530/ERC-13-0494 24521857

[6] Stenman A Zedenius J Juhlin CC . The value of histological algorithms to predict the Malignancy potential of pheochromocytomas and abdominal paragangliomas—a meta-analysis and systematic review of the literature. Cancers (Basel). (2019) 11:225. doi: 10.3390/cancers11020225 30769931 PMC6406721

[7] Wachtel H Hutchens T Baraban E Schwartz LE Montone K Baloch Z . Predicting metastatic potential in pheochromocytoma and paraganglioma: a comparison of PASS and GAPP scoring systems. J Clin Endocrinol Metab. (2020) 105:e4661–70. doi: 10.1210/clinem/dgaa608 32877928 PMC7553245

[8] Fishbein L Leshchiner I Walter V Danilova L Robertson AG Johnson AR . Comprehensive molecular characterization of pheochromocytoma and paraganglioma. Cancer Cell. (2017) 31:181–93. doi: 10.1016/j.ccell.2017.01.001 28162975 PMC5643159

[9] Martinelli S Amore F Canu L Maggi M Rapizzi E . Tumour microenvironment in pheochromocytoma and paraganglioma. Front Endocrinol (Lausanne). (2023) 14:1137456. doi: 10.3389/fendo.2023.1137456 37033265 PMC10073672

[10] Bechmann N Rosenblum JS Alzahrani AS . Current views on the role of HIF-2α in the pathogenesis and syndromic presentation of pheochromocytoma and paraganglioma. Best Pract Res Clin Endocrinol Metab. (2024) 38:101955. doi: 10.1016/j.beem.2024.101955 39426935

[11] Litwiniuk-Kosmala M Makuszewska M Czesak M . Endoglin in head and neck neoplasms. Front Med (Lausanne). (2023) 10:1115212. doi: 10.3389/fmed.2023.1115212 36844233 PMC9950573

[12] Koh JM Ahn SH Kim H Kim BJ Sung TY Kim YH . Validation of pathological grading systems for predicting metastatic potential in pheochromocytoma and paraganglioma. PloS One. (2017) 12:e0187398. doi: 10.1371/journal.pone.0187398 29117221 PMC5678867

[13] Białas M Dyduch G Dudała J Bereza-Buziak M Hubalewska-Dydejczyk A Budzyński A . Study of microvessel density and the expression of vascular endothelial growth factors in adrenal gland pheochromocytomas. Int J Endocrinol. (2014) 2014:104129. doi: 10.1155/2014/104129 25276126 PMC4167815

[14] Koo TK Li MY . A guideline of selecting and reporting intraclass correlation coefficients for reliability research. J Chiropr Med. (2016) 15:155–63. doi: 10.1016/j.jcm.2016.02.012 27330520 PMC4913118

[15] Nazari MA Rosenblum JS Haigney MC Rosing DR Pacak K . Pathophysiology and acute management of tachyarrhythmias in pheochromocytoma: JACC review topic of the week. J Am Coll Cardiol. (2020) 76:451–64. doi: 10.1016/j.jacc.2020.04.080 32703516 PMC7454044

[16] Torresan F Iacobone C Giorgino F Iacobone M . Genetic and molecular biomarkers in aggressive pheochromocytomas and paragangliomas. Int J Mol Sci. (2024) 25:7142. doi: 10.3390/ijms25137142 39000254 PMC11241596

[17] Nassiri F Cusimano MD Scheithauer BW Rotondo F Fazio A Yousef GM . Endoglin (CD105): a review of its role in angiogenesis and tumor diagnosis, progression and therapy. Anticancer Res. (2011) 31:2283–90. 21737653

[18] Ge R Wang Z Cheng L . Tumor microenvironment heterogeneity: an important mediator of prostate cancer progression and therapeutic resistance. NPJ Precis Oncol. (2022) 6:31. doi: 10.1038/s41698-022-00272-w 35508696 PMC9068628

[19] Althobiti M Muftah AA Aleskandarany MA Joseph C Toss MS Green A . The prognostic significance of BMI1 expression in invasive breast cancer is dependent on its molecular subtypes. Breast Cancer Res Treat. (2020) 182:581–9. doi: 10.1007/s10549-020-05719-x 32524353 PMC7320923

[20] Sur D Havasi A Lungulescu CV Volovat SR Burz C Irimie A . Endoglin (CD105) as a putative prognostic biomarker for colorectal cancer: a systematic review. Med Pharm Rep. (2022) 95:251–9. doi: 10.15386/mpr-2120 36060514 PMC9387571

[21] Sier VQ van der Vorst JR Quax PHA de Vries MR Zonoobi E Vahrmeijer AL . Endoglin/CD105-based imaging of cancer and cardiovascular diseases: a systematic review. Int J Mol Sci. (2021) 22:4804. doi: 10.3390/ijms22094804 33946583 PMC8124553

[22] Çordan İ Günler T . CD105 (Endoglin) expression as a prognostic marker in aggressive papillary thyroid carcinoma. Clin Endocrinol (Oxf). (2025) 103:596–604. doi: 10.1111/cen.15290 40485134 PMC12413672

[23] Milinkovic M Soldatovic I Zivaljevic V Bozic V Zivotic M Tatic S . Comprehensive investigation of angiogenesis, PASS score and immunohistochemical factors in risk assessment of Malignancy for paraganglioma and pheochromocytoma. Diagnost (Basel). (2024) 14:849. doi: 10.3390/diagnostics14080849 38667494 PMC11049119

[24] Mahaki H Nobari S Tanzadehpanah H Babaeizad A Kazemzadeh G Mehrabzadeh M . Targeting VEGF signaling for tumor microenvironment remodeling and metastasis inhibition: therapeutic strategies and insights. BioMed Pharmacother. (2025) 186:118023. doi: 10.1016/j.biopha.2025.118023 40164047

[25] Vaz Ferreira C Siqueira DR Romitti M Ceolin L Brasil BA Meurer L . Role of VEGF-A and its receptors in sporadic and MEN2-associated pheochromocytoma. Int J Mol Sci. (2014) 15:5323–36. doi: 10.3390/ijms15045323 24675699 PMC4013566

[26] Kaelin WG Jr. Von Hippel-Lindau disease: insights into oxygen sensing, protein degradation, and cancer. J Clin Invest. (2022) 132:e162480. doi: 10.1172/JCI162480 36106637 PMC9479583

[27] Pollard PJ El-Bahrawy M Poulsom R Elia G Killick P Kelly G . Expression of HIF-1alpha, HIF-2alpha (EPAS1), and their target genes in paraganglioma and pheochromocytoma with VHL and SDH mutations. J Clin Endocrinol Metab. (2006) 91:4593–8. doi: 10.1210/jc.2006-0920 16954163

[28] Cui Y Ma X Gao Y Chang X Chen S Lu L . Local-regional recurrence of pheochromocytoma/paraganglioma: characteristics, risk factors and outcomes. Front Endocrinol. (2021) 12:762548. doi: 10.3389/fendo.2021.762548 34899602 PMC8660112

[29] Luo Z Yan X Liu Y Nan F Lei Y Ren Y . Prognostic significance of Ki-67 in assessing the risk of progression, relapse or metastasis in pheochromocytomas and paragangliomas. Ann Med. (2025) 57:2478312. doi: 10.1080/07853890.2025.2478312 40079941 PMC11984564

[30] Favier J Amar L Gimenez-Roqueplo AP . Paraganglioma and phaeochromocytoma: from genetics to personalized medicine. Nat Rev Endocrinol. (2015) 11:101–11. doi: 10.1038/nrendo.2014.188 25385035

[31] Gill AJ . Succinate dehydrogenase (SDH)-deficient neoplasia. Histopathology. (2018) 72:106–16. doi: 10.1111/his.13277 29239034

[32] Jochmanová I Yang C Zhuang Z Pacak K . Hypoxia-inducible factor signaling in pheochromocytoma: turning the rudder in the right direction. J Natl Cancer Inst. (2013) 105:1270–83. doi: 10.1093/jnci/djt201 23940289 PMC3888279

[33] Takenaka J Watanabe S Abe T Hirata K Uchiyama Y Kimura R . Prognostic value of [18F]FDG-PET prior to [131I]MIBG treatment for pheochromocytoma and paraganglioma (PPGL). Ann Nucl Med. (2023) 37:10–7. doi: 10.1007/s12149-022-01798-6 36301465

