# Supplementary material for: Increased HIF-2α and CD105 (endoglin) expression is associated with poor differentiation in pheochromocytomas and paragangliomas
Source: Front Endocrinol (Lausanne). 2026 Sep 4;17:1915433. doi: 10.3389/fendo.2026.1915433 (PMC13585612; doi:10.3389/fendo.2026.1915433)
Supplement: Supplementary file 1 [file Table1.docx]

| **Supplementary Table 1. Interobserver Agreement for Immunohistochemical Assessments** | | | | |
| --- | --- | --- | --- | --- |
| **Marker** | **Agreement statistic** | **Estimate** | **95% CI** | **Interpretation** |
| HIF-2α | ICC | 0.979 | 0.964–0.986 | Excellent |
| VEGF-IRS | ICC | 0.931 | 0.880–0.966 | Excellent |
| CD105-MVD | ICC | 0.975 | 0.926–0.987 | Excellent |
| Ki-67 | ICC | 0.972 | 0.944–0.987 | Excellent |
| SDHB | Cohen κ | 1.000 | 1.000–1.000 | Perfect |
| Data are presented as ICC or Cohen’s kappa coefficient (κ), together with their 95% CIs. ICC values were calculated using a two-way mixed-effects, absolute agreement, single-measurement model. Because multiple tumor foci were available from the same patient, the confidence intervals for the ICCs were calculated using patient-level bootstrap resampling. Interobserver agreement was interpreted according to the recommendations of Koo and Li as poor (ICC <0.50), moderate (0.50–0.75), good (0.75–0.90), or excellent (>0.90) **(14).**  Abbreviations: CI, confidence interval; ICC, intraclass correlation coefficient; κ, Cohen's kappa coefficient; HIF-2α, hypoxia-inducible factor-2 alpha; VEGF, vascular endothelial growth factor; IRS, Immunoreactivity Score; MVD, microvessel density; SDHB, succinate dehydrogenase subunit B. | | | | |
