# Supplementary material for: Increased HIF-2α and CD105 (endoglin) expression is associated with poor differentiation in pheochromocytomas and paragangliomas
Source: Front Endocrinol (Lausanne). 2026 Sep 4;17:1915433. doi: 10.3389/fendo.2026.1915433 (PMC13585612; doi:10.3389/fendo.2026.1915433)
Supplement: Supplementary file 2 [file Table2.docx]

| **Supplementary table 2. Comparison of Immunohistochemical Marker Profiles According to Clinicopathological Subgroups** | | | | | | | | | |
| --- | --- | --- | --- | --- | --- | --- | --- | --- | --- |
| **Variable** | **Genetic Mutation** | | | **SDHB status** | | | **Vascular Invasion** | | |
|  | **Negative (n = 18)^1^** | **Positive**  **(n = 9)^1^** | **p^2^**  **value** | **Loss**  **(n = 4)^1^** | **Retained (n = 31)^1^** | **p^2^ value** | **Absent (n = 28)^1^** | **Present**  **(n = 7)^1^** | **p^2^ value** |
| **GAPP score** | 5.33 ± 1.53 | 6.33 ± 2.96 | 0.400 | 7.75 ± 1.71 | 5.32 ± 1.89 | 0.057 | 5.00 ± 1.63 | 8.00 ± 1.53 | **0.001** |
| **HIF-2α**  **H-score** | 78.89 ± 79.03 | 104.44 ± 63.86 | 0.400 | 142.5± 107.20 | 80.00 ± 72.94 | 0.300 | 73.57 ± 73.50 | 141.4± 77.77 | 0.067 |
| **VEGF**  **IRS score** | 3.39 ± 3.24 | 6.78 ± 2.49 | **0.007** | 5.00 ± 4.76 | 4.19 ± 3.15 | 0.800 | 4.14 ± 3.11 | 4.86 ± 4.14 | 0.067 |
| **CD105**  **MVD score** | 15.17 ± 10.38 | 29.22 ± 28.83 | 0.200 | 37.00 ± 25.42 | 19.45 ± 18.55 | 0.300 | 17.93 ± 15.86 | 35.57 ± 28.35 | 0.700 |
| **Ki-67**  **index (%)** | 2.03 ± 2.08 | 4.42 ± 3.63 | 0.100 | 3.25 ± 1.89 | 2.82 ± 3.18 | 0.700 | 2.45 ± 2.64 | 4.56 ± 4.09 | 0.200 |
| ^1^Data are presented as mean ± standard deviation. All analyses were performed on a tumor-focus basis (n = 35). Comparisons according to genetic mutation status were restricted to tumor foci from patients with available genetic testing results (n = 27 tumor foci). The mutation-positive group consisted of 9 tumor foci from 5 patients with pathogenic PPGL-associated mutations.  ^2^P-values were calculated using Welch’s t-test.  **Abbreviations:** SDHB, succinate dehydrogenase subunit B; HIF-2α, hypoxia-inducible factor-2 alpha; VEGF, vascular endothelial growth factor; IRS, immunoreactivity score; MVD, microvessel density. | | | | | | | | | |
